# Supplementary material for: Mortality rates in a cohort of infants attending immunization clinics in Uganda (2017–2019)
Source: PLoS One. 2025 May 23;20(5):e0324122. doi: 10.1371/journal.pone.0324122 (PMC12101647; doi:10.1371/journal.pone.0324122)
Supplement: S2 Data — Varible descriptions within the mortality dataset. (DOCX) [file pone.0324122.s002.docx]

| **Variable Name** | **Type** | **Length** | **Label** | **Possible Values** |
| --- | --- | --- | --- | --- |
|  |  |  |  |  |
| t1_cliniccode | Num | 8 | Clinic Code |  |
| cohort | Char | 9 | Exposure Status | Exposed  UnExposed |
| infdied | Num | 8 | Infant Died | 0=No  1=Yes |
| follow_up_time | Num | 8 | Follow up time in days |  |
| exclusivenew_ | Num | 8 | Infant breastfeeding | 1= Exclusive breastfeeding  2='Mixed' 'Replacement' |
| t1_marriage_ | Num | 8 | Mother Marital status | 1 =Living with a partner  2= Not living with partner  9=missing |
| t1_ancnum_ | Num | 8 | Gestation period at start of ANC | 1='7-9 months'  2='3-6 months'  3='0-3 months'  9=missing |
| t1_transport_ | Num | 8 | Mode of transport to Health Facility | 1= Motorized  2= Walking  9=missing |
| wealth_ses_ | Num | 8 | Social Economic Status | 1=High  2= Meduim  3= Low  9=missing |
| t1_artstart_ | Num | 8 | Mother ART Start | 1= Initiated preconception  2= Initated during pregnancy/Identified during pregnancy  3= Initiated post delivery/Identified Positive during study |
| educ_ | Num | 8 | Mother Education status | 1= Above Primary level 2='None/Primary' |
| malariapreg_ | Num | 8 | Mother had malaria in Pregnancy | 1=Yes  2=No  9= Missing |
| cohort_ | Num | 8 | Numerical variable of exposure status | 1= Exposed  0= Unexposed |
| t1_sex | Num | 8 | Sex of the child | 1=Male  2=Female |
| birthattendee_ | Num | 8 | Who attended the birth | 1= Skilled  2='Traditional Birth Attendant/Other' |
| birthplacenew_ | Num | 8 | Place of Delivery | 1=Public health facility  2= Private Health Facility  3= Home |
| baseVL | Num | 8 | Mother Baseline Viral load | 1= Suppressed  2=Non Supressed  9=missing |
| agegrp_ | Num | 8 | Mother’s categorized age group- Numeric | 1='15-24'  2= 25+ |
| agegroup | Char | 5 | Mother’s categorized age group | '15-24'  25+ |
| infageweeks | Num |  | Infant age in Weeks |  |
| t1_birthweight_i | Num | 8 | Infant birth weight |  |
| anc4plus | Num |  | Had 4 plus ANC visits | 1= Yes  2= No |
|  |  |  |  |  |
